# Best papers of 2022—Editor-in-Chief’s picks

**DOI:** 10.1093/bjsopen/zrad001

**Published:** 2023-02-13

As a new year begins, it is time to reflect what happened during 2022. Although the year has been referred by many as ‘Annus horribilis’ (horrible year) due to world events, this has not been the case for *BJS Open*. Both the quality and quantity of submissions to the journal have increased and I would like to express our gratitude to the authors and reviewers for their ever-lasting support towards the journal. While all articles published in *BJS Open* are ‘Editor’s choice’, I would like to take this moment to highlight some manuscripts for special mention that have changed my thinking.

RCTs remain one of the most powerful study types to define gold standard treatments. Despite being a relatively common condition, prospective trials regarding pilonidal disease are rare. I was delighted to see multicentre RCT from Sweden comparing midline suturing to Karydakis flap^1^. Wound healing was faster (median 49 *versus* 14 days) in patients randomized to Karydakis flap, but the recurrence rate was equally low, slightly less than 10 per cent, in both groups in a remarkable median 11-year follow-up.

Another RCT, also from Sweden, assessed the superiority of Toupet (posterior partial) to Nissen (total) fundoplication in the treatment of paraoesophageal hernias^2^. While the primary outcome (dysphagia assessed using Ogilvie score at 6 months) was not different between the groups, patients undergoing Toupet fundoplication had improved Dakkak dysphagia score and other improved swallowing functions.

Some topics are difficult or even impossible to study in a controlled prospective study. Comprehensive registry studies may provide useful data and evidence in areas where RCTs are not practical. A nationwide population-based study explored the effect of sex on survival after resection for oesophageal or gastric cancer^3^. The authors found that women had better survival after resection of oesophageal cancer compared with men. Interestingly, such a difference could not be demonstrated for gastric cancer. The reasons behind the findings are discussed in the article and are most likely multifactorial.

Concerning women’s health, breast cancer is the most common malignancy among women. Approximately 5 per cent of women seek medical treatment for infertility. As hormone therapy is an established risk factor for breast cancer, it is important to know whether fertility treatments affect breast-cancer risk. An Irish meta-analysis including 25 studies with over 600 000 patients did not find such an association and concluded that *in-vitro* fertilization does not appear to increase breast-cancer risk^4^. This is a relieving outcome for anyone needing fertility treatments.

Groin hernia is one of the most common surgical procedures worldwide and consequently even minor improvements may have huge effects at a population level. An umbrella review from Thailand assessed the current evidence regarding mesh-fixation techniques^5^. The main finding was that mesh fixation with glue leads to less chronic groin pain, which has been the Achilles’ heel of inguinal hernia repair. This benefit was noted in both open and laparoscopic repairs.

Last, but certainly not least, a review regarding posthepatectomy liver failure started a whole new article type in the journal: ‘comprehensive review’^6^. These reviews will provide a detailed evidenced-based overview of important topics for the surgical community. The reviews will serve as a starting point for new researchers as well as an update for more experienced surgeons—regardless of whether they are more academically or clinically oriented.

The *BJS Open* team hope that we can serve our readers and authors even better in 2023 and thank our invaluable peer reviewers in helping us to achieve this goal.
